# Determination of the *pK*_*a*_ Value of a Brønsted Acid by ^19^F NMR Spectroscopy

**DOI:** 10.1002/mrc.5485

**Published:** 2024-10-16

**Authors:** Emily F. Griffiths, Jay A. Dixon, Andrew J. M. Caffyn, Stuart K. Langley, Beatriz Maciá, Vittorio Caprio, Ryan E. Mewis

**Affiliations:** ^1^ Faculty of Science and Engineering, Department of Natural Sciences Manchester Metropolitan University Manchester UK

**Keywords:** ^19^F, Brønsted acids, NMR, phosphinic acids, *pK*_*a*_

## Abstract

Brønsted acids, such as phosphoric acids derived from chiral 1,1′‐bi‐2‐naphthol (BINOL), are important catalysts in the formation of carbon–carbon and carbon–heteroatom bonds, for example. The catalytic activity of these Brønsted acids is strongly linked to their acidity, and as such, the evaluation of compounds to determine *pK*_*a*_ values provides insight into their catalytic activity. Herein, a ^19^F{^1^H} NMR methodology is detailed to determine the *pK*_*a*_ of a fluorinated binaphthyl‐derived phosphinic acid, *rac‐* **1**, in acetonitrile and in the presence of a fluorinated sulfonamide reference compound (**2**–**4**). The approach was tested initially using **2** and **3**, with the Δ*pK*_*a*_ (0.08) in strong agreement with previously reported values (6.6 for **2** and 6.68/6.73 for **3**). Sigmoidal curves of normalised chemical shift change (Δδ) against equivalents of the base phosphazene P_1_‐^t^Bu added overlapped for **2** and **3**, but in the case of *rac*‐**1** and either **2**, **3** or **4**, there was significant separation. A variety of different approaches for determining the Δ*pK*_*a*_ were compared. Values of *pK*_*a*_ determined when the normalised Δδ was 90% were optimal for **2** and **3**, whereas a normalised Δδ of 75% was optimal for **4**, resulting in the *pK*_*a*_ of *rac*‐**1** being determined to be 8.47–8.71.

## Graphical abstract

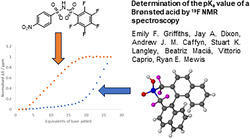

## 1 Introduction

**FIGURE 1:**
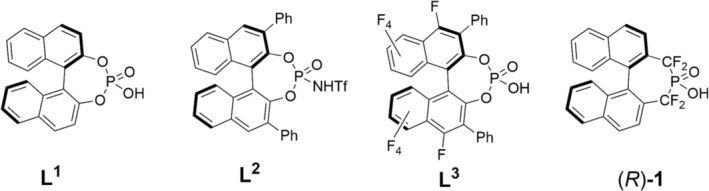
*R*‐enantiomers of a binaphthyl‐derived chiral phosphoric acid (**L** ^**1**^), *N*‐triflylphosphoramides (**L** ^**2**^), binaphthyl‐derived chiral phosphoric acid with perfluorobiaryl framework (**L** ^**3**^) and a perfluoroalkyl binaphthyl‐derived chiral phosphinic acid ((*R*)‐**1**).

A number of reactions are catalysed by chiral Brønsted acids, which are, largely, binaphthol‐based (e.g., **L** ^**1**^–**L** ^**3**^ in Figure 1) [1, 2, 3]. The catalytic activity of Brønsted acid ligands is linked to their acidity, and, as such, there is a need to develop more strongly acidic catalysts to broaden the scope of enantioselective transformations. The modification of **L** ^**1**^ (Figure 1), a 1,1′‐bi‐2,2′‐napthol (BINOL)‐derived chiral phosphoric acid, can lead to stronger Brønsted acids. Enhanced acid acidity of **L** ^**1**^ can be obtained by modification of either the acid moiety (e.g., **L** ^**2**^) [4] or the chiral backbone (e.g., **L** ^**3**^) [5, 6, 7].

With respect to the modification of the acid moiety, one way to increase the acidity of any given phosphoric acid catalyst is to incorporate fluorine at the α‐position relative to the phosphorous atom [8]; (*R*)‐**1** exemplifies this approach. Indeed, in the 1980s, it was postulated that to create phosphate mimics based on phosphonates, α‐halogenation and, more specifically, α‐fluorination of the phosphonates, were required [9, 10]. The resulting *pK*_*a*_ of the singularly or doubly fluorinated phosphonates was comparable to that of phosphate [8]. Use of perfluoroalkyl groups has also been utilised to produce more sterically demanding phosphoric and phosphinic acids for organocatalysis, to achieve better enantioselectivity [6, 7, 11]. Furthermore, it has been reported that the acidity of phosphonic acids bearing a difluoromethylene group at the α‐position of the phosphorus atom was more acidic than the parent phosphoric acid [8].

Fujii et al. [7] have reported a series of perfluoroalkyl phosphinic acids, namely, (*R*)‐**1** and two further derivatives where a single fluorine of each CF_2_ group is replaced by a CF_3_ or C_2_F_5_ group. The perfluoroalkyl groups are not only electron‐withdrawing, thus leading to a stronger acid, but also possesses helical chirality. Improved enantioselectivity for the Friedel‐Crafts reaction of *N*‐tosylimine with indole was obtained with increasingly bulkier fluoroalkyl groups in the order C_2_F_5_ > CF_3_ > F (76%, 66.5% and 54.5% *ee*, respectively). The yields obtained also increased when using bulkier substituents from 56% for F to 89% for C_2_F_5_. The *pK*_*a*_ values for the acids utilised were not reported. Obtaining *pK*_*a*_ values can provide further insight into the catalytic activity of the phosphinic acids (and other Brønsted acids) under scrutiny, crucially evidencing how catalyst structure is linked to increased acidity. This approach should enable the production of better catalysts.

A number of studies have been conducted, whereby the *pK*_*a*_ values of acids have been determined. For example, the acidity scale of strong, neutral Brønsted acids has been determined using UV–vis spectroscopy [12]. The *pK*_*a*_ value is determined from the mean Δ*pK*_*a*_ value obtained from numerous spectra after the addition of titrant to two acids [13]. The use of this spectrophotometric method, coupled with rate constants for the Nazarov cyclisation of a dienone, showed that there was a direct correlation between Brønsted acidity and rate of reaction, in that stronger acids led to increased catalytic activity [14]. Furthermore, it was deduced that the acids studied could be classified into three distinct groups, based on their *pK*_*a*_ range: binaphthyl‐derived phosphoric acids (*pK*_*a*_ range = 12–14), fluorinated *N*‐sulphonylphosphoramides (*pK*_*a*_ range 6–7) and bis (sulfuryl)imides (*pK*_*a*_ = *~*5) [15]. In 2021, a revised *pK*_*a*_ scale consisting of 231 acids and spanning almost 30 orders of magnitude was published [12]. The *pK*_*a*_ values are linked by 569 Δ*pK*_*a*_ measurements. The gas phase acidities of 29 compounds, inclusive of aromatic sulphonimides, have also been experimentally determined using a Fourier transform ion cyclotron resonance equilibrium measurement approach and compared to their *pK*_*a*_ values [16]. Similarly, the *pK*_*a*_ values of substituted sulphonamides in acetonitrile‐water binary mixtures by UV–visible spectroscopy have also been reported [17].

There are alternatives to the well‐established spectrometric approach to determine *pK*_*a*_ values. NMR spectroscopy has been employed to determine the *pK*_*a*_s of 26 fluorinated carboxylic acids over the pH range 0.3–10 [18]. Titration curves were established by using ^19^F NMR spectra collected at 17 different pH vales. Good agreement was obtained between values determined and those fluorinated acids for which *pK*_*a*_ values were already known. Shivapurkar and Jeannerat [19] have also described an approach for the determination of *pK*_*a*_ values that is based on NMR spectroscopy. Aliased ^1^H‐^13^C HSQC spectra were used to determine the *pK*_*a*_ values of acids such as coumaric and camphanic acid. The approach involves the titrations of two acids simultaneously; one of the acids is the reference, for which the *pK*_*a*_ is known and from which the *pK*_*a*_ of the other acid can be determined based on the chemical shifts obtained. The chemical shifts required are obtained from three conditions: two for the fully protonated and deprotonated forms of each acid and another one where both acids are partially protonated. ^13^C NMR data were acquired, as it is more sensitive, in terms of chemical shift changes, than ^1^H NMR spectroscopy. However, implementation of HSQC was required to offset the intrinsic insensitivity associated with acquiring ^13^C NMR data, whilst also enabling the drift of many signals to be tracked simultaneously.

**FIGURE 2:**
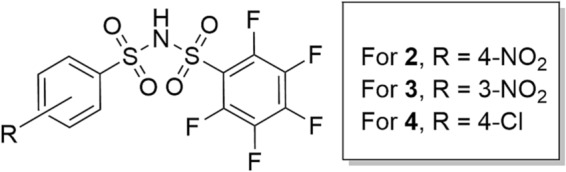
Chemical structures of reference Compounds **2**–**4**.

Herein, we showcase an NMR titration method that utilises the large chemical shift window of ^19^F to determine the *pK*_*a*_ of *rac*‐**1**, using benzenesulphonamides as reference compounds (**2**–**4**, Figure 2). Unlike ^13^C, ^19^F has a similar sensitivity to that of ^1^H, and therefore, there is no requirement for 2D spectroscopic methods. ^19^F NMR spectroscopy is sensitive to changes in pH provided the ^19^F nuclei are near an acid site. This is aptly demonstrated in reports that showcase the development and employment of pH‐responsive sensors and indicators to monitor chemical or biological change [20, 21, 22, 23].

## 2 Results and Discussion

### 2.1 Synthesis of *rac*‐1 and Reference Compounds 2–4

The synthesis of (*R*)‐**1** has been reported previously by Fujii et al. [7]; a modified approach was utilised herein for the synthesis of *rac*‐**1** (see the Supporting Information). The three reference compounds, **2**–**4**, were synthesised according to, or adapted from, reported literature procedures [12, 16, 24]. Single crystals suitable for X‐ray crystallographic analysis were obtained for *rac*‐**1**, its ethyl precursor **7**, starting material **5** and two of the reference compounds (**3** and **4**). Their structures are reported in the Supporting Information.

### 2.2 Preliminary Testing of the ^19^F{^1^H} NMR Titration Method to Determine the *pK*_*a*_ of Reference Standards

Prior to determining the *pK*_*a*_ value of the Brønsted acid *rac*‐**1**, a control ^19^F{^1^H} NMR titration was performed using two of the reference sulfonamides compounds, **2** and **3**, for which the *pK*_*a*_ values are known; the *pK*_*a*_ of **2** is 6.60 [12], whereas **3** has a *pK*_*a*_ of 6.68 [12] or 6.73 [16]. These two reference standards were chosen due to the similarity in their chemical shifts compared to **4** (Figure S1). Thus, being able to determine the *pK*_*a*_ of either acid would demonstrate the applicability of the method under development with respect to determining the *pK*_*a*_ of *rac*‐**1**.

To ascertain the difference in *pK*_*a*_ via ^19^F{^1^H} NMR spectroscopy, an equimolar amount of compounds **2** and **3** were fully protonated using an excess of a super acid, neat triflic acid. Subsequently, **2** and **3** were deprotonated, by the addition of aliquots of the strong base *tert*‐butylimino‐tris (dimethylamino)phosphorane (P_1_‐^*t*^Bu). For each aliquot of base added, a ^19^F{^1^H} NMR spectrum was acquired. The titration plot is shown in Figure S2. From these spectra, a difference in *pK*_*a*_ was calculated as 0.08 using equations outlined previously [19]. In brief, the observed values for both acids were taken as close as possible to where the normalised signal intensity is 50% of its maximum. This small difference in Δ*pK*_*a*_ is expected given the similarity in the two sigmoidal curves obtained for both acids; the larger the difference in *pK*_*a*_, the larger the separation will be between the two sigmoidal‐curves.

A plot of the acid–base pairs, for two acids, *i* and *j*, should lie on a straight line:

(1) $$x=\left({\delta_{j}}^{\mathrm{obs}}-{\delta_{j}}^{B-}\right)\left({\delta_{i}}^{B–H}-{\delta_{i}}^{\mathrm{obs}}\right),y=\left({\delta_{i}}^{\mathrm{obs}}-{\delta_{i}}^{B-}\right)\left({\delta_{j}}^{B–H}-{\delta_{j}}^{\mathrm{obs}}\right),$$

which require the chemical shifts at the observed (obs) titration point, fully protonated (B‐H) and fully deprotonated (B−). For the titration of **2** and **3**, the plot of these *x* and *y* coordinates is shown in Figure S3. The logarithm of the gradient of the straight line is equal to the Δ*pK*_*a*_. This gives a value of Δ*pK*_*a*_ of 0.079 ± 0.011. The error was calculated using a Jack–Knife approach [25, 26]. Both values obtained show good agreement and they also compare well with the literature values.

We note that despite using equimolar amounts of **2** and **3**, it was not always possible to obtain integral ratios of 1:1 as the signals partially overlapped over the course of the titration. Thus, to obtain such a small difference in *pK*_*a*_ was pleasing, especially given the expected Δ*pK*_*a*_ range of 0.08 to 0.13 (based on the two different reported values for **3**). Given this result, we moved on to evaluate the *pK*_*a*_ of *rac*‐**1**.

**FIGURE 3:**
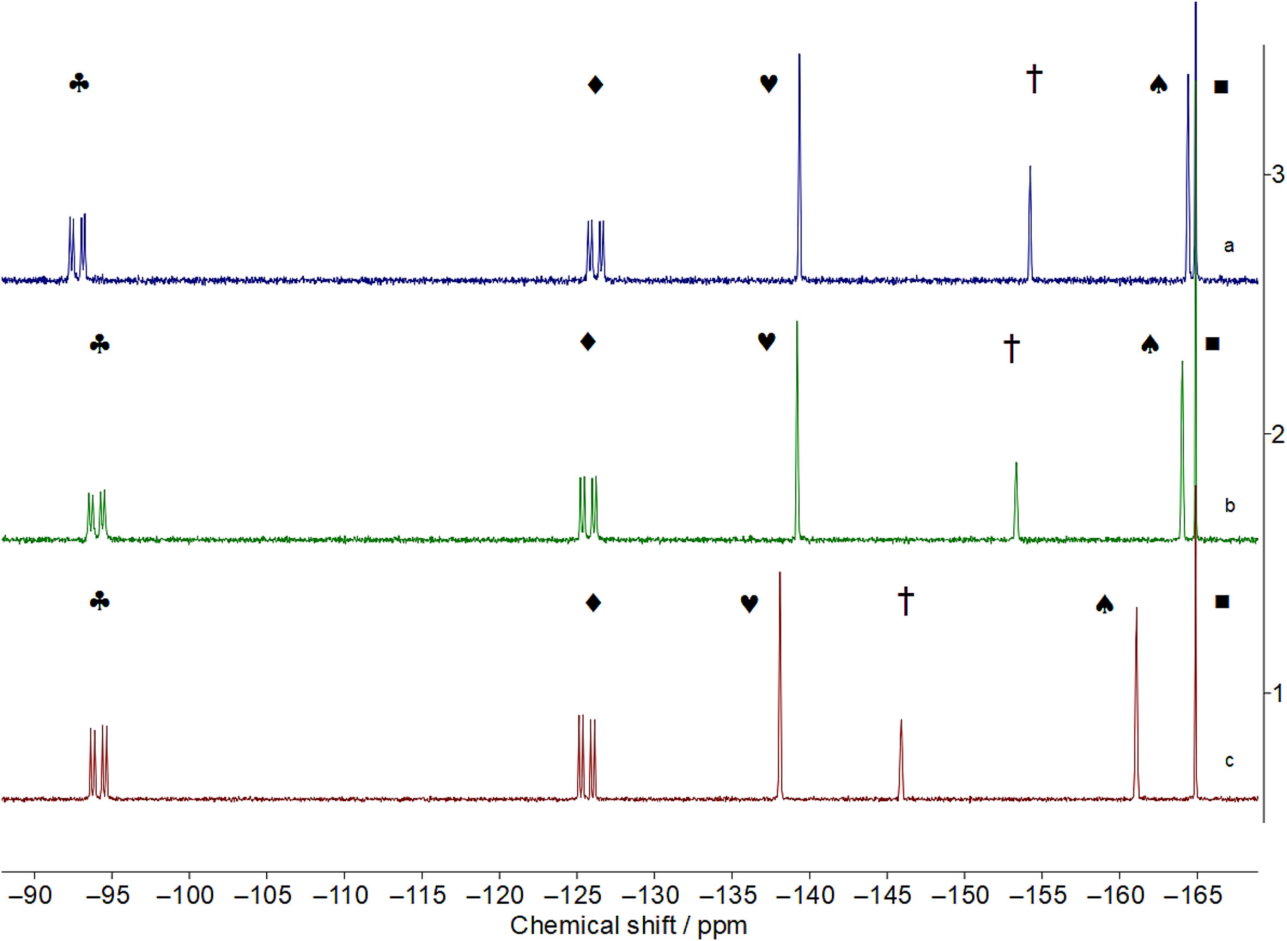
Exemplar ^19^F{^1^H} NMR spectra for the reaction of the acid forms of equimolar amounts of *rac*‐**1** and **2** (Spectrum 1), in the presence of hexafluorobenzene (▪), to the fully deprotonated forms (Spectrum 3). Spectrum 2 is a midpoint between the fully deprotonated and protonated forms. Symbols ♣ and ♦ belong to *rac*‐**1**, whereas the three signals labelled ♥, † and ♠ belong to **2**.

To calculate the *pK*_*a*_ value of *rac*‐**1**, first, ^19^F{^1^H} NMR spectra of *rac*‐**1** with one of the reference compounds (**2**–**4**) were obtained when fully protonated, fully deprotonated and partially protonated/deprotonated. Hexafluorobenzene was used as an internal standard (^19^F NMR signal at −164.90 ppm), which was added to the sample in a sealed tube. An example of this approach is shown in Figure 3 for an equimolar amount of *rac*‐**1** and **2**.

Figure 3 shows the effect of the addition of phosphazene P_1_‐^*t*^Bu on *rac*‐**1** and **2**. The ^19^F{^1^H} NMR signals of *rac*‐**1**, in acidic conditions, are located at −94.15 and −125.63 ppm (symbols ♣ and ♦, respectively). These signals shift to −93.88 and −126.23 ppm, respectively, in the fully deprotonated form of **1**. Similarly, the ^19^F{^1^H} NMR signals of **2**, located at −138.11 (♥, *ortho*), −145.91 (†, *para*) and −162.35 (♠, *meta*), shift to −139.18, −153.33 and −164.04 ppm, respectively, in the fully deprotonated form. The three ^19^F{^1^H} NMR spectra presented in Figure 3 represent the start of the titration (protonated), a middle point and the end of the titration (deprotonated). These data are sufficient to calculate the difference in acidity constants as 1.31, from which the *pK*_*a*_ of *rac*‐**1** is deduced to be 7.91, when using the chemical shifts of the most downfield ^19^F NMR signal of *rac*‐**1** and the *meta* ^19^F NMR signal of **2**. Furthermore, the *pK*_*a*_ value derived is based on **2** having a *pK*_*a*_ of 6.60 in MeCN and also being the more acidic (shown to deprotonate first in the ^19^F NMR titration; see later). Using the *para* or *ortho* ^19^F NMR signal of *rac*‐**1** to perform the same calculation yielded similar *pK*_*a*_ values of 7.92 and 7.93, respectively, for *rac*‐**1**. It is noteworthy that the titration of **1** in the presence of **3** and **4** was also completed. When **3** was used, the *pK*_*a*_ range of **1** was found to be 8.13–8.16, so consistent with those obtained when **2** was used, whereas employment of **4** yielded a *pK*_*a*_ range for **1** of 8.59–8.62. The differences in the *pK*_*a*_ values determined for **1** then became the focus of further investigation.

**FIGURE 4:**
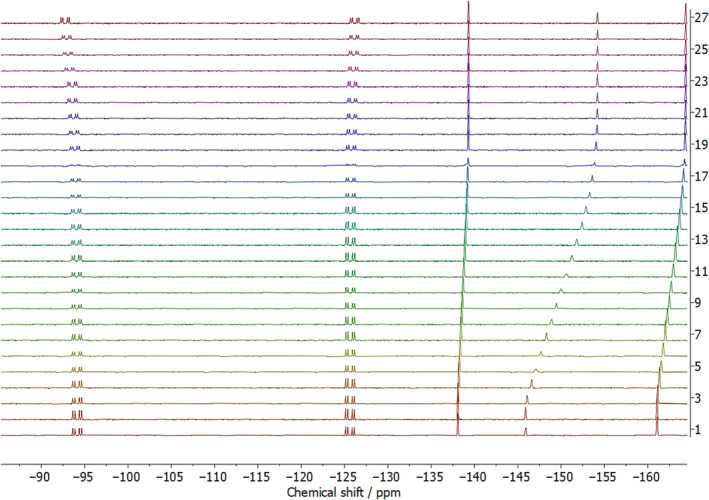
Stacked ^19^F{^1^H} NMR spectra results for the titration of *rac*‐**1** using reference Compound **2**. The starting point for the titration is Spectrum 1, whereas the end‐point is Spectrum 27.

The approach of obtaining the chemical shifts for the protonated and deprotonated forms of *rac*‐**1** and one of the reference compounds, as well as a third point for when the compounds are partially deprotonated, is adequate to calculate the *pK*_*a*_, as demonstrated above. However, a more robust approach is to collect a series of data points over the entire titration range; Shivapurkar and Jeannerat [19] demonstrate this via a titration consisting of 25 points. However, it is notable that the solubility of *rac*‐**1** in acetonitrile is very low, and this means that 64 transients are required to obtain a ^19^F NMR spectrum with appropriate signal‐to‐noise that can be integrated. The ^19^F NMR titrations reported herein are, therefore, quite costly in terms of time, with each titration point taking around 7 min of instrument time to acquire. Nonetheless, the advantages of *pK*_*a*_ determination by titration overcome the time‐consuming nature of the process, as outlined by Shivapurkar and Jeannerat [19]. The results from the ^19^F{^1^H} NMR titration of **1** using reference Compound **2** is shown in Figure 4 as an example.

Prior to starting the titration, a spectrum was acquired to ensure the phosphinic acid *rac*‐**1** and reference Compound **2** were within 1% integral size of each other (integrals should be 1:1) prior to fully protonating the samples to ensure equal molarities were present in the sample. The concentration of the sample is not required to calculate the change in *pK*_*a*_ as long as the molar amounts are equal.

Spectrum 1 of the stacked plot shown in Figure 4 represents the starting point of the titration. The data from spectrum 1 also accounted for the observed values, $\delta_{i}^{\mathrm{obs}}$ and $\delta_{j}^{\mathrm{obs}}$ for the *pK*_*a*_ calculation, where *i* and *j* represent phosphinic acid *rac*‐**1** and reference Compound **2**, respectively. Each subsequent spectrum, 2–27, correlates to the addition of 20 μL P_1_‐^*t*^Bu in acetonitrile solution (4.56 mM) until the end point was reached, and both compounds were fully deprotonated (Spectrum 29, Figure 4). The peak intensities decrease after each addition of base. This is due to the samples being effectively diluted during the course of the experiment.

**FIGURE 5:**
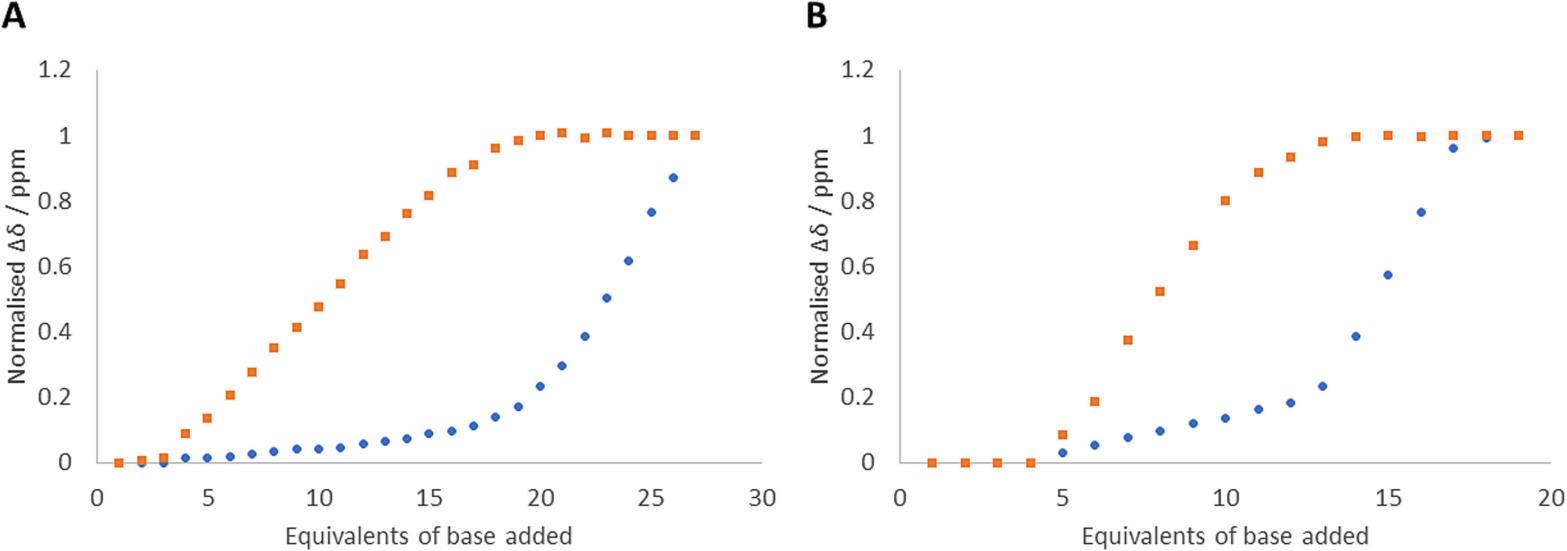
Sigmoidal curves of the normalised chemical shifts of the reference compound (orange squares, *A* = **2** and *B* = **4**) and *rac*‐**1** (blue circles).

In terms of deducing the Δ*pK*_*a*_ between the reference and the acid, a number of different approaches were employed. This is important when the *pK*_*a*_ values of the reference acids are considered; although the *pK*_*a*_s of **2** and **3** are similar, they are dissimilar to that of **4**, as they differ by approximately one *pK*_*a*_ unit (*pK*_*a*_ of **4** = 7.55 [12]). This affects the sigmoidal‐curves obtained, as shown in Figure 5A,B, in that the curves are closer together for *rac*‐**1** and **4** compared to *rac*‐**1** and **2**. This infers that the true *pK*_*a*_ of *rac*‐**1** is closer to that of **4** than to **2** (and by extension, **3**). Another point to make is that the reference has fully deprotonated prior to *rac*‐**1** becoming no more than 25% deprotonated. This has implications in terms of the points used when employing Equation (1) as when the reference is fully deprotonated, δ_j_^obs^ = δ_j_^B−^, and results in one set of coordinates becoming zero.

Shivapurkar and Jeannerat show that optimal conditions for determining the Δ*pK*_*a*_ are dependent on the *pK*_*a*_ difference between the reference and the acid [19]. When the difference is small, that is, Δ*pK*_*a*_ = 0.1, then the observed values for the reference and acid should be taken as close as possible to when the value of the normalised Δδ is 50% of the maximum values. This changes to 75%/25% and 90% /10%, respectively, for the observed values of the reference and acid for *pK*_*a*_ differences of 1 and 2. The higher values in each pairing are for the material that reaches the fully deprotonated step first.

The data reported in Table 1 showcase Δ*pK*_*a*_ when the data obtained are scrutinised differently. First, the average of chemical values was taken until the slope in the sigmoidal‐curve for *rac*‐**1** became more vertical than horizontal; this is referred to as *pK*_*a*(*average*)_. The cut‐off point was an arbitrarily chosen point. Second, the same process was completed but only used values until 90% of the maximum reference normalised Δδ was obtained, which is referred to as *pK*_*a*(*average*, 90%)_. The difference between these two sets of values is fairly minimal but does highlight that there is a stark difference in Δ*pK*_*a*_s between the nitro‐substituted reference acids (**2** and **3**) and **4**. The difference is consistent, regardless of how the data are scrutinised. Third, a single point was taken where the normalised Δδ is 90% of its maximum for the reference acid and 10% for *rac*‐**1** (*pK*_*a*(*single point*, 90%)_). This process was repeated for the fourth set of data values, but this time the threshold for the normalised Δδ was set at 75% of its maximum for the reference acid and 25% for *rac*‐**1** (*pK*_*a*(*single point*, 75%)_).

**TABLE 1:** *pK*_*a*_ values determined for *rac*‐**1** in acetonitrile and in the presence of Reference Compounds **2**–**4** when examined using differing sampling conditions.

Reference	*pK*_*a*(*average*)_	*pK*_*a*(*average*, 90%)_	*pK*_*a*(*single point*, 90%)_	*pK*_*a*(*single point*, 75%)_
**2**	7.9088	7.9193	8.4661	8.2246
**3**	8.1301	8.1725	8.5832	8.3014
**4**	8.5967	8.5967	9.2746	8.7070

The third and fourth set of data values in Table 1 show the greatest disparity. However, the difference in *pK*_*a*_ of the different reference acids must be considered, using that of the sigmoidal curves obtained as reference. Thus, it can be stated that the Δ*pK*_*a*_ is smaller when *rac*‐**1** is titrated in the presence of **4** compared to **2** and **3** reference compounds. Three of the four methods indicate the *pK*_*a*_ of *rac*‐**1** in the presence of **4** as being between 8.60 and 8.71 (Table 1). The outlier, *pK*_*a*(*single point*, 90%)_, is due to the fact that when the normalised reference Δδ is ~90%, the Δδ of *rac*‐**1** is *~*15–16%, thus entailing the value obtained is nonideal. These data suggest a Δ*pK*_*a*_ difference of less than 2 between *rac*‐**1** and **4** and imply that the *pK*_*a*(*single point*, 75%)_ is the most accurate (Δ*pK*_*a*_ of ~1). Conversely, the lower *pK*_*a*_ values of **2** and **3** (6.6–6.73) result in a higher Δ*pK*_*a*_ difference of ~2 *pK*_*a*_ units. Therefore, the *pK*_*a*(*single point*, 90%)_ will be the most accurate for these two reference acids. Overall, the *pK*_*a*_ of *rac*‐**1** can be determined to be 8.47–8.71, which utilises the *pK*_*a*(*single point*, 90%)_ for **2** and **3** and the *pK*_*a*(*single point*, 75%)_ for **4**. When compared to the known *pK*_*a*_ values of the reference compounds used, the *pK*_*a*_ range determined for *rac*‐**1** reflects a Δ*pK*_*a*_ of approximately two for the two regioisomer reference compounds (**2** and **3**) and one for **4**. In all cases, the reference compounds deprotonate first, implying that *rac*‐**1** is a weaker acid in comparison. However, the *pK*_*a*_ range determined for *rac*‐**1** means it is a stronger acid, by an order of four or more *pK*_*a*_ units, when compared to other BINOL phosphoric acids [12]. The acidity of *rac*‐**1** is comparable to *p*‐toluenesulfonic acid (*pK*_*a*_ = 8.5 in MeCN) and benzenesulfonic acid (*pK*_*a*_ = 8.2 in MeCN) but significantly less acidic compared to *N*‐sulfonyl phosphoramides (*pK*_*a*_ = *ca*. 6–7) and sulfonyl imides (*pK*_*a*_ = *ca*. 5) [15].

## 3 Conclusions

The *pK*_*a*_ of phosphinic acid *rac*‐**1** was determined using three synthesised sulfonamide reference compounds, **2**–**4**, via ^19^F{^1^H} NMR‐based titrations. Using acetonitrile as a solvent, *rac*‐**1** and either **2**, **3** or **4** were treated with triflic acid (to ensure complete protonation) prior to titration using phosphazene base P_1_‐^*t*^Bu as the titrant. The resulting sigmoidal curves were utilised to obtain Δ*pK*_*a*_ values. Initially, a pair of reference acids (**2** and **3**) were analysed to ensure that the Δ*pK*_*a*_ was consistent with values from existing literature. The Δ*pK*_*a*_ for reference Compounds **2** and **3** was shown to be *~*0.08, which correlated well published *pK*_*a*_ values for the compounds (6.6 for **2** and 6.68/6.73 for **3**).

The *pK*_*a*_ of *rac*‐**1** was determined to be 8.47–8.71 using reference Compounds **2**–**4**. When scrutinising these data, the difference in *pK*_*a*_ values between that of *rac*‐**1** and each reference compound must be considered, and this is reflected in the distance between the sigmoidal curves obtained; the greater the distance, the greater the Δ*pK*_*a*_. Thus, *pK*_*a*(*single point*, 90%)_ values were used for reference Compounds **2** and **3**, whereas the *pK*_*a*(*single point*, 75%)_ value was used for **4**. The *pK*_*a*_ range determined for *rac*‐**1** reflects a Δ*pK*_*a*_ of approximately two for the two regioisomer reference compounds (**2** and **3**) and one for **4**. The *pK*_*a*_ of *rac*‐**1** suggests that this acid has similar acidity to *p*‐toluenesulfonic acid (*pK*_*a*_ = 8.5 in MeCN) and benzenesulfonic acid (*pK*_*a*_ = 8.2 in MeCN).

## Supporting information


**Figure S1.** Stacked 19F NMR spectra of reference sulfonamides synthesised, compounds 2, 3 and 4 (A‐C respectively). Symbols †, ♦ and ♠ denote the ortho‐, para‐ and meta‐fluorine signals respectively. Data collected in DMSO‐d6.
**Table S1.** 19F chemical shift values used for the fully protonated and fully deprotonated forms of rac‐1 relative to the reference standards 2–4 in the 19F{1H} NMR titration experiments performed.
**Figure S2.** Normalised change in chemical shift for reference compounds 2 (blue circles) and 3 (orange circles) plotted as a function of titrant added.
**Figure S3.** Plot of (δjobs − δjB−)(δiB–H − δiobs) (x) against (δiobs – δiB−)(δjB–H – δjobs) (y) for the titration of 2 and 3 using phosphazene base P1‐tBu.
**Figure S4.** Ball and stick representations of the crystal structures of rac‐1 (A) and 7 (B). Atom labelling:carbon (black), hydrogen (white), oxygen (red), fluorine (pink) and phosphorous (blue).
**Table S2.** Selected geometric parameters for the crystal structures of rac‐1 and 7.
**Figure S5.** Ball and stick representation of the crystal structure of 5. Atom labelling:carbon (black) and hydrogen (white).
**Figure S6.** Ball and stick representation of the X‐ray crystallographic structure of 3 that showcases the coordination to sodium ions by ligand molecules in lattice. Atom labelling:carbon (black), oxygen (red), nitrogen (blue), fluorine (pink), sulphur (yellow) and sodium (silver).
**Figure S7.** Ball and stick representation of the X‐ray crystallographic unit cell contents of 4 with H atoms omitted for clarity. For atoms that are disordered over two positions, only one position is shown. The sodium ion in the centre of the unit cell has half occupancy compared to the other four sodium ions shown. Atom labelling:carbon (black), oxygen (red), nitrogen (blue), fluorine (pink), sulphur (yellow) and sodium (silver).
**Figure S8.** Ball and stick representation of the X‐ray crystallographic unit cell contents of 4 viewed along the b‐c plane. H atoms have been omitted for clarity. For atoms that are disordered over two positions, only one position is shown. Atom labelling:carbon (black), oxygen (red), nitrogen (blue), fluorine (pink), sulphur (yellow) and sodium (silver).
**Table S3.** Crystallographic data for compounds rac‐1, 3–5 and 7.
