# Supplementary material for: Determination of the pK a Value of a Brønsted Acid by 19F NMR Spectroscopy
Source: Magn Reson Chem. 2024 Oct 16;63(1):17–23. doi: 10.1002/mrc.5485 (PMC11608935; doi:10.1002/mrc.5485)
Supplement: Supplementary file 1 — Figure S1. Stacked 19F NMR spectra of reference sulfonamides synthesised, compounds 2, 3 and 4 (A‐C respectively). Symbols †, ♦ and ♠ denote the ortho‐, para‐ and meta‐fluorine signals respectively. Data collected in DMSO‐d6. Table S1. 19F chemical shift values used for the fully protonated and fully deprotonated forms of rac‐1 relative to the reference standards 2–4 in the 19F{1H} NMR titration experiments performed. Figure S2. Normalised change in chemical shift for reference compounds 2 (blue circles) and 3 (orange circles) plotted as a function of titrant added. Figure S3. Plot of (δjobs − δjB−)(δiB–H − δiobs) (x) against (δiobs – δiB−)(δjB–H – δjobs) (y) for the titration of 2 and 3 using phosphazene base P1‐tBu. Figure S4. Ball and stick representations of the crystal structures of rac‐1 (A) and 7 (B). Atom labelling:carbon (black), hydrogen (white), oxygen (red), fluorine (pink) and phosphorous (blue). Table S2. Selected geometric parameters for the crystal structures of rac‐1 and 7. Figure S5. Ball and stick representation of the crystal structure of 5. Atom labelling:carbon (black) and hydrogen (white). Figure S6. Ball and stick representation of the X‐ray crystallographic structure of 3 that showcases the coordination to sodium ions by ligand molecules in lattice. Atom labelling:carbon (black), oxygen (red), nitrogen (blue), fluorine (pink), sulphur (yellow) and sodium (silver). Figure S7. Ball and stick representation of the X‐ray crystallographic unit cell contents of 4 with H atoms omitted for clarity. For atoms that are disordered over two positions, only one position is shown. The sodium ion in the centre of the unit cell has half occupancy compared to the other four sodium ions shown. Atom labelling:carbon (black), oxygen (red), nitrogen (blue), fluorine (pink), sulphur (yellow) and sodium (silver). Figure S8. Ball and stick representation of the X‐ray crystallographic unit cell contents of 4 viewed along the b‐c plane. H atoms have been omitted for [file MRC-63-17-s001.docx]

**Determination of the pK_a_ value of a Brønsted acid by ^19^F NMR spectroscopy**

Emily Griffiths†, Jay Dixon†, Andrew Caffyn, Stuart Langley, Beatriz Macia-Ruiz, Vittorio Caprio, Ryan E. Mewis*

^1^Faculty of Science and Engineering, Department of Natural Sciences, Manchester Metropolitan University, Chester Street, Manchester, UK. M1 5GD

† These authors contributed equally to this manuscript

^*^Corresponding author(s): Dr Ryan E. Mewis (email. [r.mewis@mmu.ac.uk](mailto:r.mewis@mmu.ac.uk))

# Contents

[Contents 1](#_Toc175234913)

[1. Experimental 2](#_Toc175234914)

[2. Stacked ^19^F{^1^H} NMR spectra of the three reference compounds 10](#_Toc175234915)

[3. Plots of equivalents of titrant added against change in chemical shift 12](#_Toc175234916)

[4. Plots of (δ_j_^obs^ − δ_j_^B−^)(δ_i_^B–H^ − δ_i_^obs^) against (δ_i_^obs^ – δ_i_^B−^)(δ_j_^B–H^ – δ_j_^obs^)for different reference acid pairings 13](#_Toc175234917)

[5. Background theory for the determination of ΔpK_a_ values 14](#_Toc175234918)

[6. Crystal structures of *rac*-**1**, **5** and **7** 15](#_Toc175234919)

[7. Crystal structures of **3** and **4** 18](#_Toc175234920)

[8. References 22](#_Toc175234921)

# Experimental

## Chemicals

All materials for synthetic procedures were purchased from Fischer Scientific, Sigma Aldrich or Strem Chemicals. All materials were used as received unless otherwise stated. The solvents used were of general purpose or HPLC grade and were purchased from Fisher Scientific. TLC analysis was performed using aluminium-backed silica gel 60 F254, 0.2 (Merck plates) or aluminium-backed aluminium oxide 60 F254, (Merck plates). Silica gel chromatography was performed with silica gel 60 (Davisil). Alumina gel chromatography was performed using aluminium oxide 150 (Aldrich). When required, diethyl ether, dichloromethane and acetonitrile were dried as follows; diethyl ether was dried over sodium metal and benzophenone followed by distillation; dichloromethane and acetonitrile were dried over phosphorus pentoxide for 24 h followed by distillation.

## Instrumentation

Unless stated otherwise, all reactions were carried out in inert conditions under either argon or nitrogen using standard Schlenk techniques. All reagents used were of analytical grade and used as received from suppliers, unless stated otherwise. Anhydrous solvents and chemicals were dried and obtained using a Pure Solvent cabinet. The triethylamine used was dried and stored over 3 Å molecular sieves for prolonged storage. The acetonitrile used was distilled and stored over phosphorus pentoxide for prolonged storage. All TLC experiments used plates coated in silica powder and compounds purified by column chromatography used a tall column of silica gel; shorter columns (5 cm) were used for flash chromatography purification. NMR spectra were acquired on either a JEOL ECS 400 MHz FT NMR spectrometer or a JEOL ECA 500 MHz FT NMR spectrometer. Melting points were obtained using a Cole-Parmer melting point apparatus.

## ^19^F{^1^H} NMR titrations

All NMR spectra were obtained from a JEOL ECS 400 MHz FT NMR spectrometer. A single pulse experiment was altered for the optimised ^19^F{^1^H} NMR spectra titration experiments. The sweep width was set to 200 ppm and offset to −150 ppm. Each spectrum was acquired using 16384 points, 64 scans and 2 prescans. The flip angle was set to 30° and a relaxation delay of 3 seconds was used. The optimised titration was conducted under an argon atmosphere using Kontes taps to establish the inert atmosphere. The Kontes tap was fitted with a rubber septum to enable the addition of base throughout the titration while maintaining the inert atmosphere for the duration of the titration. The solvent used for all pK_a_ titrations was deuterated acetonitrile (CD_3_CN). The concentration of the reference compounds **2** and **3** was 9.90 x10^-3^ M and 10.6 x10^-3^ M for **4**. The concentration of the fluorinated phosphinic Brønsted acid (*rac*-**1**) was 10.3 x10^-3^ M. The superacid used to fully protonate all species prior to the titration was a solution of triflic acid in CD_3_CN (100 µL, 0.063 M). The strong base used throughout the titrations was a solution of phosphazene base P_1_-^t^Bu in CD_3_CN (0.021 M) in 20 µL additions per spectrum recording/titre. An internal standard solution of hexafluorobenzene in CD_3_CN (7.18 x10^-3^ M) was used as a reference point for each titration spectrum and was sealed into a melting point tube to ensure the pK_a_ titration results were unaffected.

## X-ray data collection and structural refinement

Single crystal X-ray diffraction data were collected on a Rigaku Saturn724+ CCD diffractometer, using either MoKα X-rays of λ=0.71073 Å or CuKα X-rays of λ=1.541840 Å. When MoK was employed as the radiation source, crystals were cooled to 150 K during data collection.

Refinement of F^2^ was performed against all reflections. The weighted R-factor, wR, and goodness of fit, S, are based on F^2^, conventional R-factors (R) are based on F, with F set to zero for negative F^2^. The threshold expression of F^2^ > 2sigma(F^2^) is used only for calculating R-factors(gt) etc. and is not relevant to the choice of reflections for refinement. R-factors based on F^2^ are statistically about twice as large as those based on F, and R-factors based on ALL data will be even larger.

Structures presented were solved by direct methods. All non-H atoms were refined anisotropically. Hydrogen atoms were fixed in idealised positions and refined using a riding model, with C-H distances of 0.97 Å, N-H distances of 0.91 Å, and U_iso_ 1.5 times U_eq_ of the carrier atom. All Ortep representations show ellipsoids at the 50% probability level.

The data collection, cell refinement and data reduction was conducted using CrysAlisPro.^1^ The solution and refinement of the data was achieved using SHELXL-97.^2^

## Synthesis of phosphinic acid *rac-***1**

The phosphinic acid *rac-***1** was synthesised according to Scheme S1 which is modified from an existing literature procedure reported by Mikami *et al.*^3^ Crystal structures of *rac-***1**, **5** and **7** were obtained and are detailed in section 6 of this SI.


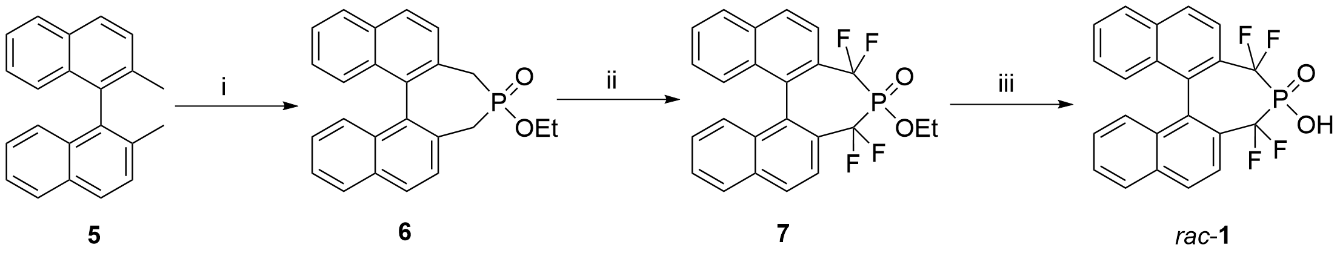


Scheme S1: Synthesis of *rac-***1**. Conditions: i) n-BuLi, TMEDA, −72°C, 30 min then ethyldichlorophosphate, 0°C, 30 min; ii) NaHMDS, *N*-fluorobenzenesulfonimide, −79°C to RT, 2 hr; iii) TMSBr, Δ, 96 hr.

### Synthesis of 2,2’-dimethyl-1,1’-binaphthyl (**5**)

To a stirred solution of 2,2’-bistriflate-1,1’-binaphthyl (5.00 g, 9.1 mmol) and Ni(dppp)Cl_2_ (0.21 g, mmol) in Et_2_O (30 mL) was added MeMgI (12 mL, 3.0 M in Et_2_O) dropwise at 0°C. Reaction mixture was heated to reflux for 12 hours. The reaction mixture was quenched on ice with 1 M HCl and washed with EtOAc. Water was added and the mixture was neutralised using K_2_CO_3_. The organics were dried over MgSO_4_, filtered and solvent removed under reduced pressure to afford the product as a white solid (1.10 g, 43%); ^1^H NMR (500 MHz, CDCl_3_) δ 7.90 (s, 4H), 7.50 (s, 2H), 7.40 (s, 2H), 7.20 (s, 2H), 7.04 (s, 2H), 2.03 (s, 6H); ^13^C{^1^H} NMR (125.77 MHz, CDCl_3_) δ 135.0, 134.4, 132.9, 132.4, 128.9, 128.1, 127.6, 126.2, 125.8, 125.4, 20.1.

### Synthesis of ethyl-2,2’-bis(methylene)-1,1’-binaphthyl phosphinate (**6**)

To a solution of **5** (0.0788 g, 0.28 mmol) in dry THF (2 mL) was added *n*-butyllithium (2.23 M in hexanes, 2.4 eq.) at -72°C. The reaction mixture was stirred for 10 minutes at the same temperature before the addition of TMEDA (1.1 eq. to n-butyllithium, 0.74 mmol, 0.1 mL). The reaction mixture was stirred for 30 minutes before allowing to warm to room temperature, until the mixture had completed a colour change to crimson red. At 0°C, ethyldichlorophosphate (33 µL, mmol) was added and the reaction left to stir for 30 minutes. Reaction mixture was quenched with saturated NH_4_Cl at 0°C, CH_2_Cl_2_ (50 mL) was then added to the reaction vessel and separated. The aqueous layer was washed a further three times with CH_2_Cl_2_ (3 x 50 mL) and the organics were combined and dried using MgSO_4_, prior to filtration by gravity. The solvent was removed under reduced pressure to afford the crude product. The crude product was purified by flash column chromatography, eluting with EtOAc to afford a white solid (0.0333 g, 32%); ^1^H NMR (500 MHz, CDCl_3_) δ 7.89 (t, *J* = 15.76 Hz, 4H), 7.51 (d, J = 10.00 Hz, 2H), 7.39 (m, J = 15.0 Hz, 2H), 7.21 (m, J = 15.0 Hz, 2H), 7.05 (d, J = 8.50 Hz, 2H), 4.10 (m, 1H), 3.64 (t, 1H) 1.32 (m, 3H). ^31^P{^1^H} NMR (202.47 Hz, CDCl_3_) δ 57.47; ^13^C{^1^H} NMR (125.77 MHz, CD_2_Cl_2_) δ 134.0 (dd, J = 28 Hz, J = 5 Hz), 133.1 (d, J = 16 Hz), 131.2 (d, J = 10 Hz), 130.1 (d, J = 9 Hz), 129.4 (d, J = 18 Hz), 128.6 (d, J = 9 Hz), 128.3 (dd, J = 28 Hz, J = 5 Hz), 127.1 (d, J = 8 Hz), 126.6, 125.9, 61.1 (d, J = 7 Hz), 34.8 (dd, 1 JC-P = 88 Hz, J = 67 Hz), 16.9 (d, 5 Hz); HRMS calcd. for C_24_H_21_NaO_2_P^+^ [(M+Na)^+^]: 395.1171 found: 395.1189.

### Synthesis of **7**

A solution of **6** (2.68 g, 7.2 mmol) in THF (100 mL) was added, over a period of 3 mins, to a solution of NaHMDS (7.27 g, 39.7 mmol) in THF (100 mL) at -78 °C. The resulting orange/red solution was stirred for 1 h at -78°C and a solution of *N*-fluorobenzenesulfonimide (16.59 g, 52.6 mmol) in THF (100 mL) was added over a period of 3 mins. The reaction was stirred at -78 °C for a further 2 h. The reaction was allowed to warm up to room temperature over a period of 5 mins and quenched with 0.01 N aq. HCl (100 mL). The reaction mixture was then reduced *in vacuo*. The mixture was extracted with EtOAc (3 × 100 mL) and combined, prior to being dried over MgSO_4_. The solution was then concentrated under reduced pressure before being purified by flash chromatography over silica gel (CHCl_3_ then CHCl_3_/EtOAc (98:2)) to the title compound as a cream coloured crystalline material (1.93 g, 60%); m.p: 90-93°C; R_f_: 0.32 (CHCl_3_); IR (KBr pellet, cm^-1^): 3066 (CH aromatic), 2986 (CH aliphatic), 1596 and 1508 (CC aromatic), 1273 (P=O), 1100 and 1066 (CF), 1015 and 949 (POC), 857 and 816 (PC aliphatic), 747 (CH aromatic); ^1^H NMR (400 MHz, CD_2_Cl_2_): δ 8.18 (t, J = 9 Hz, 2H, Ar), 8.03 (d, J = 8 Hz, 2H, Ar), 7.85 (dd, J = 21 Hz, J = 9 Hz, 2H, Ar), 7.60 (t, J = 7 Hz, 2H, Ar), 7.32 (t, J = 8 Hz, 2H, Ar), 7.14 (d, J = 9 Hz, 2H, Ar), 4.43 (m, 2H, P-O-CH_2_- ), 1.38 (t, J = 7 Hz, 3H, -CH_3_); ^13^C{^1^H} NMR (100 MHz, CD_2_Cl_2_): δ 134.9, 132.9, 130.5, 130.4, 128.8, 128.3, 127.7, 127.4, 126.9 (br s), 121.8 (dd, J = 27 Hz, J = 14 Hz), 118.3 (dt, 1 J_C-P_ = 138 Hz, 1 J_C-F_ = 271 Hz, -CF_2_-P). ^19^F{^1^H} NMR (CD_2_Cl_2_): δ -91.5 (dd, ^2^J_F-F_ = 277 Hz, ^2^J_F-P_ = 87 Hz, 1F), -93.5 (dd, ^2^J_F-F_ = 295 Hz, ^2^J_F-P_ = 110 Hz, 1F), -121.7 (ddd, ^2^J_F-F_ = 283 Hz, ^2^J_F-P_ = 98 Hz, ^4^J_F-F_ = 12 Hz, 1F), -123.8 (ddd, ^2^J_F-F_ = 294.8 Hz, ^2^J_F-P_ = 93 Hz, ^4^J_F-F_ = 12 Hz, 1F); ^31^P{^1^H} NMR (CD_2_Cl_2_): δ 25.6 (pseudo-pent, ^2^J_P-F_ = 105 Hz, ^2^J_P-F_ = 92 Hz); HRMS (ESI) calcd. for C_24_H_17_F_4_NaO_2_P^+^ ([M+Na]^+^) = 4467.0795, found = 467.0793.

### Synthesis of *rac*-**1**

TMSBr (0.92 mL, 1.07 g, 6.99 mmol) was added to a solution of **7** (1.50 g, 3.38 mmol) in CH_2_Cl_2_ (50 mL) at room temperature. This was heated to reflux for 96 h (with TLC monitoring). A second aliquot of TMSBr (0.45 mL, 0.52 g, 3.41 mmol) was added and refluxed until complete silylation is achieved (≈ 8 h). Solvent and un-reacted TMSBr were removed *in vacuo*. The resulting pale brown residue was then dissolved in MeOH (60 mL) and stirred at room temperature for 24 h. Solvent was removed under reduced pressure to give bis(difluoromethylene)-1,1’- binaphthyl phosphinic acid as a pale brown powder (1.39 g, 99%). ^1^H NMR (DMSO-d_6_): δ 11.04 (br s, 1H, P-OH), 8.18 (d, J = 9 Hz, 2H, Ar), 8.08 (d, J = 8 Hz, 2H, Ar), 7.75 (d, J = 9 Hz, 2H, Ar), 7.56 (t, J = 8 Hz, 2H, Ar), 7.32 (d, J = 7 Hz, 2H, Ar), 6.99 (d, J = 9 Hz, 2H, Ar); ^13^C{^1^H} NMR (DMSO-d_6_): δ 133.7, 133.6, 132.2, 129.6, 129.2 (br s), 128.6, 127.5, 127.4, 126.7, 122.0, 119.2 (dt, ^1^J_C-P_ = 137 Hz, ^1^J_C-F_ = 269 Hz, -CF_2_-P); ^19^F{^1^H} NMR (376 MHz, DMSO-d_6_): δ -90.6 (dd, ^2^J_F-F_ = 280 Hz, ^2^J_F-P_ = 87 Hz, 2F), -124.1 (dd, ^2^J_F-F_ = 283 Hz, ^2^J_F-P_ = 87 Hz, 2F); ^31^P{^1^H} NMR (DMSO-d_6_): δ 18.1 (pent, ^2^J_P-F_ = 87 Hz); HRMS (ESI) calcd. for C_22_H_12_F_4_O_2_P^−^ ([M]^−^ ) = 415.0511, found = 415.0522.

## Synthesis of reference compounds

Reference compounds **2**-**4** were synthesised according to Scheme S2 and were adapted from existing literature procedures.^4-6^ Crystal structures of **3** and **4** were obtained and are detailed in section 7 of this SI.


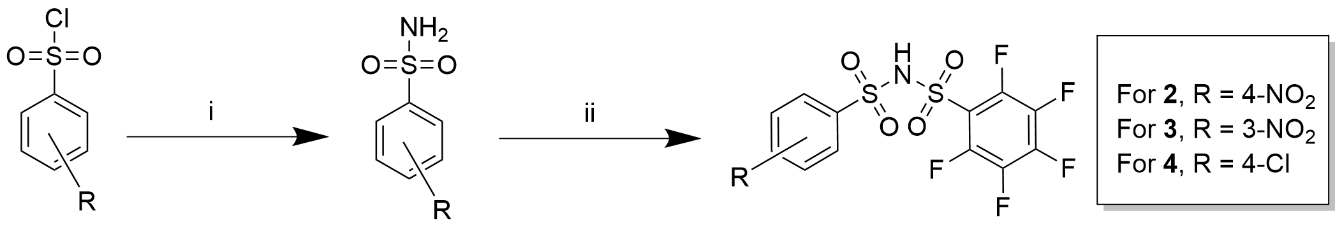


Scheme S2: Synthesis of the reference compounds **2**-**4**. Conditions: i) NH_4_OH, reflux, 5 hr; ii) NaOH 1 hr.

### General procedure for the amination of sulfonyl chlorides

Solid sulphonyl chloride derivative (1 g, 4.5 mmol) was dissolved in an excess of ammonium hydroxide and heated to reflux until completion, monitored by TLC analysis. The reaction mixture was left to cool and the resulting precipitate was filtered over vacuum and washed with ice cold water to obtain pure sulphonamide derivative (up to 94%).

### Amination of 4-nitrobenzenesulphonyl chloride (adapted from ref.^5^)

4-nitrobenzenesulphonyl chloride (1.0337 g, 4.66 mmol) was dissolved in ammonium hydroxide (8 mL) and heated to reflux overnight. On cooling, a yellow slurry was formed and the mixture was filtered. The solid was washed with water (1 mL) to obtain pure 4-nitrobenzenesulphonamide (0.7605 g, 80%); ^1^H NMR (400 MHz, DMSO-*d*_6_) δ 8.38 (d, *J* = 8.7 Hz, 2H), 8.02 (d, *J* = 8.7 Hz, 2H), 7.70 (s, 2H).

### Amination of 3-nitrobenzenesulfonyl chloride (adapted from ref.^5^)

3-Nitrobenzenesulfonyl chloride (1.0071 g, 4.54 mmol) was dissolved in ammonium hydroxide (8 mL) and the solution was heated to reflux for 5 hours. The reaction mixture was left to cool overnight to room temperature. White crystals of 3-nitrobenzenesulfonamide were obtained by filtering the slurry (0.8610 g, 94%). ^1^H NMR (400 MHz, DMSO-*d*_6_) δ 8.59 (t, *J* = 2.1 Hz, 1H), 8.46 (d, *J* = 8.0 Hz, 1H), 8.24 (d, *J* = 8.0 Hz, 1H), 7.89 (t, *J* = 8.0 Hz, 1H), 7.68 (br s, 2H); ^13^C{^1^H} NMR (100 MHz, DMSO-*d*_6_) δ 147.7, 145.6, 131.7, 131.1, 126.5, 120.6.

### Amination of *p*-chlorobenzene sulfonyl chloride^5^

*p*-Chlorobenzene sulfonyl chloride (2.0282 g, 10.58 mmol) was dissolved in ammonium hydroxide (32 mL) and stirred at 30°C for 3 hours. The reaction mixture was left to cool slowly to room temperature before isolating the product by filtration. *p*-Chlorobenzene sulfonamide was obtained as a white solid and was washed using ice-cold hexane before being dried over P_2_O_5_ for a week (1.35 g, 61%); ^1^H NMR (400 MHz, DMSO-*d*_6_) δ 7.82 (d, *J* = 8.6 Hz, 2H), 7.66 (d, *J* = 8.6 Hz, 2H), 7.47 (s, 2H).

### Synthesis of 2,3,4,5,6-pentafluoro-N-(4-nitrobenzene-1-sulfonyl)benzene-1-sulfonamide (**2**)

Synthesis adapted from Leito *et al.*^4, 6^

4-nitrobenzenesulphonamide (0.5565 g, 2.75 mmmol) was dissolved in NaOH (5%, 2.3 mL). The solution was heated to 50-60°C and pentafluorosulphonyl chloride (0.7852 g, 2.95 mmol) was added over 1 hour. The temperature and pH were monitored throughout. A pale-yellow slurry was obtained and the reaction was left to stir at the same temperature for a further 20 minutes, prior to the addition of 1 M NaOH (1.5 mL). Reaction completion was ascertained using both ^19^F NMR and ^1^H NMR spectroscopy. The slurry was filtered to obtain the crude product. The crude product was dissolved in the minimum amount of water and heated to 40-50 °C for better dissolution. Insoluble solids were removed by filtering the solution. The filtrate was acidified using concentrated HCl. The solution was transferred to a petri dish to recrystalise by evaporation. Before complete evaporation, the remaining solution was filtered and the crystals were rinsed with the minimum amount of ethanol, before being dissolved in water and left to evaporate to afford white crystals (0.0286 g, 13%); ^1^H NMR (400 MHz, DMSO-*d*_6_) δ 8.33 – 8.24 (m, 2H), 7.94 – 7.87 (m, 2H); ^19^F{^1^H} NMR (376 MHz, DMSO-*d*_6_) δ −137.44 (d, *J* = 22.6 Hz, *o*-ArF, 2F), −151.05 – −151.60 (m, *p*-ArF, 1F), -161.38 – -163.19 (m, *m*-Ar_F_, 2F); LC-MS (Q-TOF, ESI, neg.) calcd. for C_12_H_5_F_5_N_2_O_6_S_2_ ([M-H]^+^) = 430.9431, found = 430.9454, R_t_ = 4.268.

### Synthesis of 2,3,4,5,6-pentafluoro-N-(3-nitrobenzene-1-sulfonyl)benzene-1-sulfonamide (**3**)

Synthesis adapted from Leito *et. al.*^4, 6^

3-Nitrobenzenesulfonamide (0.5113 g, 2.53 mmol) was dissolved in NaOH (5%, 2.3 mL) and heated to 55°C. F_5_C_6_SO_2_Cl (0.6770 g, 2.54 mmol) was added in small portions over 1.5 hours. The reaction mixture was filtered to afford the crude product. The crude product was dissolved in a minimum amount of hot water and left to cool to room temperature to afford colourless crystals (0.3 g, 27 %); ^1^H NMR (DMSO-d_6_) δ 8.33-8.38 (m, 2H), 8.07-8.11 (m, 1H), 7.72-7.81 (m. 2H); ^19^F{^1^H} NMR (376 MHz, DMSO-*d*_6_) δ −137.50 (d, *J* = 25.6 Hz, *o*-ArF, 2F), −151.26 (m, *p*-ArF, 1F),−161.41 – −162.46 (m, *m*-Ar_F_, 2F); ^13^C{^1^H} NMR (101 MHz, CD_3_CN) δ 147.7, 145.6, 131.7, 131.1, 126.5, 120.5; ^13^C{^19^F} NMR (101 MHz, CD_3_CN) δ 129.6 (d, J = 6.14), 128.3 (d, J = 6.38), 125.2-125.1 (m), 123.8-123.7 (m), 122.1; LC-MS (QTOF, ESI, neg.) calcd. for C_12_H_4_F_5_N_2_O_6_S_2_ ([M-H]^+^) = 430.9431, found =430.9454, R_t_ = 4.272.

### Synthesis of 2,3,4,5,6-pentafluoro-N-(4-chloro-1-sulfonyl)benzene-1-sulfonamide (**4**)

Synthesis adapted from Leito *et. al.*^4, 6^

4-chlorobenzenesulfonamide (1.3498 g, 7.043 mmol) was dissolved in NaOH (5%, 6 mL) and heated to 63°C. Pentafluorobenzenesulfonylchloride (2.0765, mmol) was added in small portions over 1 hour. The pH was maintained between 9-11 by addition of 5% NaOH as necessary. The reaction mixture was stirred for a further 25 minutes at the same temperature and left to stir overnight at 56°C. The reaction mixture was cooled slowly to room temperature and a white solid precipitated formed. The white solid was collected by filtration to afford the crude product. The crude product was recrystallized from the minimum amount of MeOH and conc. HCl (1:4) to afford white crystals (0.15 g, 5%); ^1^H NMR (400 MHz, DMSO-*d*_6_) δ 7.62 (d, *J* = 8.5 Hz, 2H), 7.48 (d, *J* = 8.6 Hz, 2H), 3.40 (s, NH, 1H); ^13^C{^1^H} NMR (101 MHz, DMSO-*d*_6_) δ 143.4, 135.6, 128.4, 128.2; ^19^F{^1^H} NMR (376 MHz, CD_3_CN) δ −138.51-(−138.46) (m, *o*-Ar F, 2F), −152.98 (*p*-Ar F, 1F), −163.56-(−163.45) (*m*, 2F, m-Ar F); LC-MS (QTOF, ESI) calcd. for C_12_H_5_Cl_1_F_5_N_2_O_4_S_2_Na_1_ ([M+Na]^+^) = 443.9166, found = 443.9160, R_t_ = 12.922.

# Stacked ^19^F{^1^H} NMR spectra of the three reference compounds


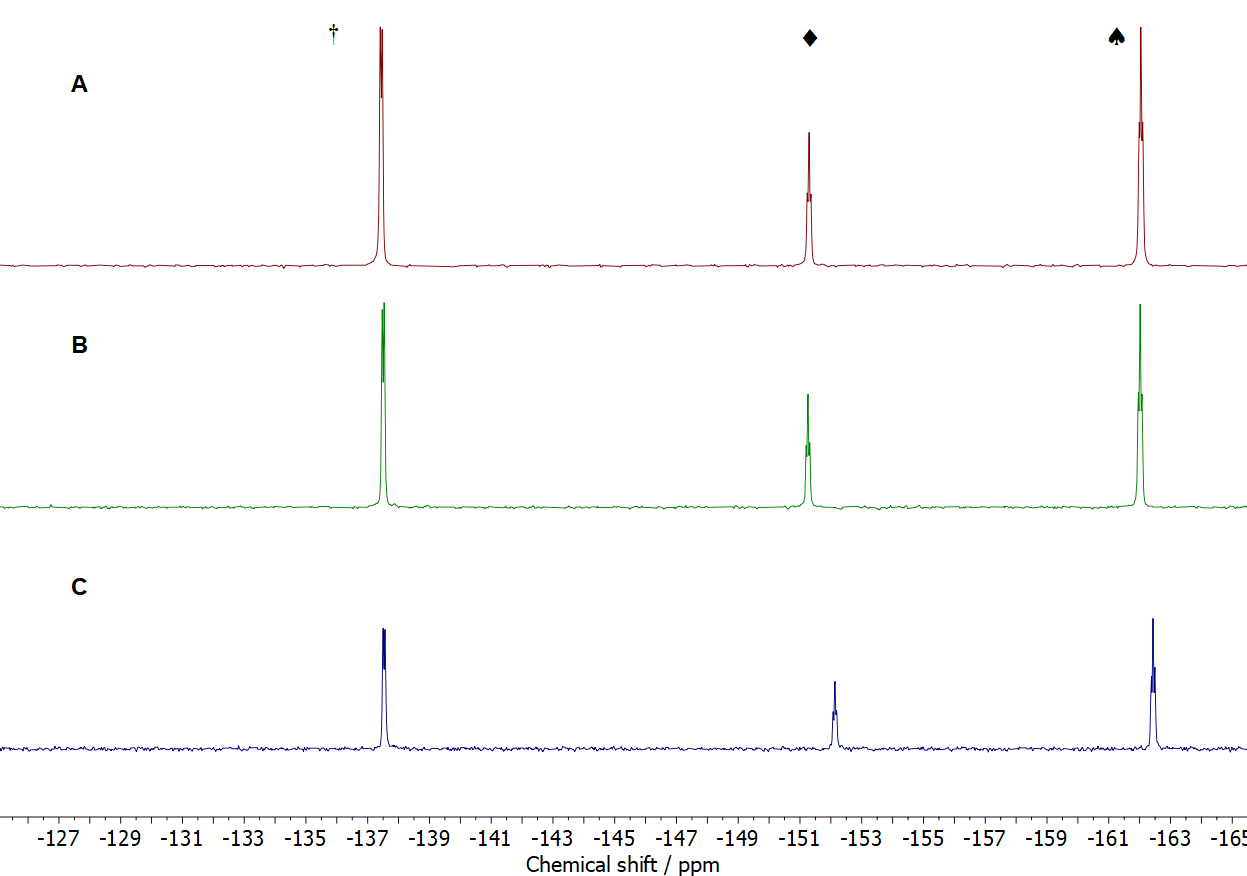


Figure S1: Stacked ^19^F NMR spectra of reference sulfonamides synthesised, compounds **2**, **3** and **4** (A-C respectively). Symbols †, ♦ and ♠ denote the *ortho*-, *para*- and *meta*-fluorine signals respectively. Data collected in DMSO-d_6_.

Table S1 – ^19^F chemical shift values used for the fully protonated and fully deprotonated forms of *rac*-**1** relative to the reference standards **2**-**4** in the ^19^F{^1^H} NMR titration experiments performed

|  | Compound **2** (ortho) | Compound **2** (para) | Compound **2** (meta) | Compound rac-**1** up-field peak | Compound rac-**1** downfield peak |
| --- | --- | --- | --- | --- | --- |
| Fully protonated | −138.08 | −145.92 | −161.08 | −125.63 | −94.14 |
| Fully deprotonated | −139.33 | −154.23 | −164.39 | −126.14 | −92.78 |
|  | Compound **3** (*meta*) | Compound **3** (*para*) | Compound **3** (*ortho*) | Compound *rac*-**1** up-field peak | Compound *rac*-**1** downfield peak |
| Fully protonated | −138.11 | −146.01 | −161.31 | −125.51 | −94.07 |
| Fully deprotonated | −139.22 | −154.38 | −164.66 | −126.21 | −92.80 |
|  | Compound **4** (*meta*) | Compound **4** (*para*) | Compound **4** (*ortho*) | Compound *rac*-**1** up-field peak | Compound *rac*-**1** downfield peak |
| Fully protonated | −138.01 | −146.15 | −161.11 | −125.47 | −93.99 |
| Fully deprotonated | −139.19 | −154.76 | −164.57 | −126.08 | −92.51 |

# Plots of equivalents of titrant added against change in chemical shift

Figure S2: Normalised change in chemical shift for reference compounds **2** (blue circles) and **3** (orange circles) plotted as a function of titrant added

# Plots of (δ_j_^obs^ − δ_j_^B−^)(δ_i_^B–H^ − δ_i_^obs^) against (δ_i_^obs^ – δ_i_^B−^)(δ_j_^B–H^ – δ_j_^obs^)for different reference acid pairings

Figure S3: Plot of (δ_j_^obs^ − δ_j_^B−^)(δ_i_^B–H^ − δ_i_^obs^) (x) against (δ_i_^obs^ – δ_i_^B−^)(δ_j_^B–H^ – δ_j_^obs^) (y) for the titration of **2** and **3** using phosphazene base P_1_-^t^Bu.

The logarithm of the slope returns the ΔpKa as being 0.08. The reported pK_a_ values for **3** are 6.68^6^ or 6.73^4^ whilst **2** is 6.6.^6^

# Background theory for the determination of ΔpK_a_ values

The pK_a_ calculation is based on the determination of the ratio of the fully protonated/deprotonated forms of the acid/base pair, where p is the proportion of the protonated form (Equation 1), as reported by Shivapurkar and Jeannerat.^7^

$r=\frac{p}{1-p}$ Equation 1

The acidity constant, K_a_ (Equation (2), can be expressed as a function of r

$K_{a}=\frac{\left| B^{-} \right|\left| H^{+} \right|}{\left| BH \right|}$ Equation 2

$K_{a}=\frac{\left| H^{+} \right|}{r}$ Equation 3

In this case, a reference compound is used and thus the mixture of compounds requires consideration of the equality (Equation 4):

$K_{a_{i}}r_{i}= K_{a_{j}}r_{j}$ Equation 4

This equality holds true for any pair of acids and bases i and j and results to (Equation 5):

$\frac{K_{a_{i}}}{K_{a_{j}}}=\frac{\left( \delta_{j}^{obs}- \delta_{j}^{B^{-}} \right)\left( \delta_{i}^{B-H}- \delta_{i}^{obs} \right)}{\left( \delta_{i}^{obs}- \delta_{i}^{B^{-}} \right)\left( \delta_{j}^{B-H}- \delta_{j}^{obs} \right)}$ Equation 5

The difference in acidity constants provided can then be used to determine the pK_a_ of the potential Brønsted acid catalyst according to Equation 6:

$\Delta pK_{a_{ij}}=\log(\frac{K_{a_{i}}}{K_{a_{j}}})$ Equation 6

The pK_a_ values of **2**-**4** in MeCN are known.^4, 6^ Prior to the pK_a_ determination of **1**, the pK_a_s of the reference compounds was determined and validated against the literature values.

# Crystal structures of *rac*-**1**, **5** and **7**

Crystals of *rac*-**1**, and its corresponding ethyl ester, **7** and dimethyl BINOL (**5**, an intermediate in the synthesis of *rac*-**1**) were obtained. *rac*-**1** and **7** both crystallised in the P_21_/_c_ space group with 8 molecules in the unit cell; their crystal structures are shown in Figure S4 in addition to selected geometric parameters which are given in Table S2. These geometric values show only slight differences compared to the analogous phosphoric acid structure (O instead of CH_2_F of *rac*-**1**), **8**.^8^ The C_napthyl_-C_napthyl_ bond elongates in **7** and *rac*-**1** to 1.5053 and 1.4954 Å, respectively, compared to **8** which is 1.4844 Å. The P-OR (R = H or Et) and P=O bonds in **7** and *rac*-**1** are both longer compared to **8** by 0.01-0.04 Å and 0.01-0.03 Å respectively. The fluorine bond lengths in **1** and **7** are slightly longer (ca. 0.03 Å (averaged)) for the latter compared to the former.


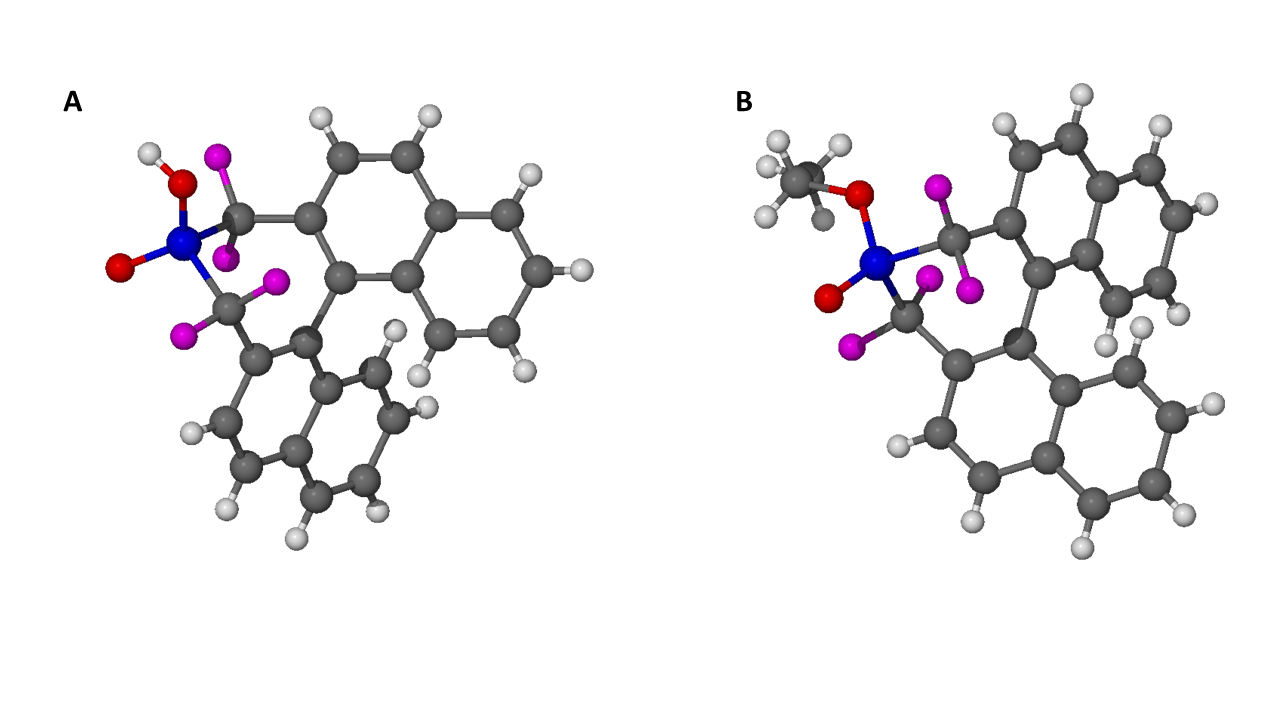


Figure S4: Ball and stick representations of the crystal structures of *rac*-**1** (A) and **7** (B). Atom labelling: carbon (black), hydrogen (white), oxygen (red), fluorine (pink) and phosphorous (blue).

Table S2: Selected geometric parameters for the crystal structures of *rac*-**1** and **7**.

| Bond | rac**-1** / Å | **7** / Å |
| --- | --- | --- |
| C_napthyl_-CF_2_P | 1.4974 and 1.5040 | 1.5097 and 1.5108 |
| C_napthyl_-C_napthyl_ | 1.4954 | 1.5053 |
| C-F | 1.3708, 1.3677, 1.3662 and 1.3702 | 1.3746, 1.3755, 1.3753 and 1.3754 |
| C-P | 1.8650 and 1.8712 | 1.8902 and 1.8736 |
| P–OR | 1.5383 (R=H) | 1.5643 (R= ethyl) |
| P=O | 1.4863 | 1.4645 |

The torsion angle between the two naphthalene moieties is 67.6° and 68.6° for the two crystallographically independent molecules of *rac*-**1**, whereas for **7** the angles are 65.3° and 66.5°. In all cases, the torsion angle was measured using the four carbons of the naphthalene rings that are involved in the seven membered ring. The dihedral angle for **8** between the planes of the naphthalene rings is reported to be 61.1°.^8^ As the C_napthyl_-CH_2_P and C-P bond lengths are both considerably longer in *rac*-**1** and **7** compared to the analogous C_napthyl_-O and C_napthyl_O-P bonds of **8**, the seven membered rings of *rac*-**1** and **7** are less constrained, and hence a higher dihedral angle is observed.

Comparison of the X-ray crystallographic structure of *rac*-**1** with a fluorinated phosphinic methyl ester analogue, which possesses two CF(C_2_F_5_) groups instead of CF_2_ groups (**9**),^3^ the torsion angles are comparable (65.6° for **9** compared to an average of 68.1° for *rac*-**1**). The slightly smaller torsion angle possessed by **9** may be due to the bulky perfluoroalkyl groups, which are in close proximity to the reaction site, leading to compression. The bond lengths between atoms of the seven-membered ring are very similar for *rac*-**1**, **7** and **9** (1.5 Å), as is the P=O bond lengths (**9** = 1.46 Å). Further, the P-OR bond lengths of **7** and **9** (1.56 Å) are consistent with one another. Thus, it is only the torsion angle of the seven membered ring that is affected by the nature of the substituent on the methylene carbons.


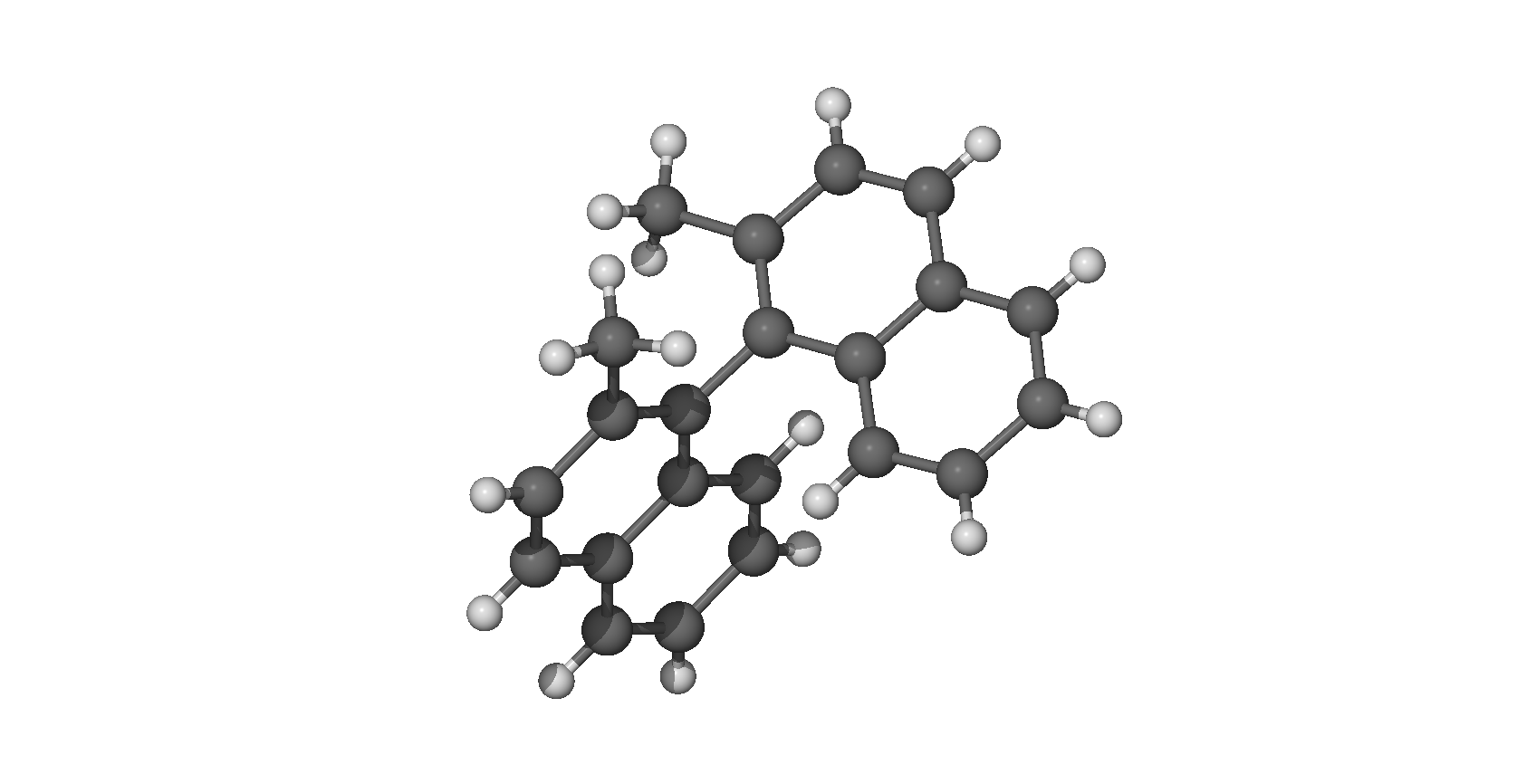


Figure S5: Ball and stick representation of the crystal structure of **5**. Atom labelling: carbon (black) and hydrogen (white).

The crystal structure of **5** was also obtained (Figure S5). Although reported previously in the literature,^9^ the crystal structure obtained herein has crystallised in a different space-group (C_2_/_c_ instead of P2_1_) and consists of eight molecules in the unit cell (instead of four). Compared to *rac*-**1** and **7**, the torsion angle between the two naphthalene rings increases to −92.9°, which correlates strongly with the reported torsion angles of 89.6° and 81.9°.^9^ The increased torsion angle, relative to *rac*-**1** and **7** is due to the lack of a seven membered ring constraining the rings. As such, the naphthalene rings are almost perpendicular to one another.

# Crystal structures of **3** and **4**

Compound **3** crystallised in the space group P-1. Sodium ions are coordinated to four different ligands and two water molecules. The water molecules are arranged *cis* to one another. The H_2_O-Na-OH_2_ bond angle is 89.35°. Bond angles for the three *trans* O-Na-O arrangements are 154.53°, 171.75° and 176.58°. The two bond angles that are closer to 180° both involve a water molecule. The sodium ion is again in a distorted octahedral geometry.

In the crystal structure, π-π stacking is visible when viewed along vector a. The centroid-to-centroid distance, linked by symmetry code x-1, y, z, between phenyl rings of the same molecule is 3.576 Å. The same distance is again observed between phenyl rings of adjacent molecules in the lattice.

Interestingly, the sulphonate group oxygens, with respect to their coordination to sodium ions, alternate between both being both involved or only one of the atoms being involved. This is shown in Figure S6.


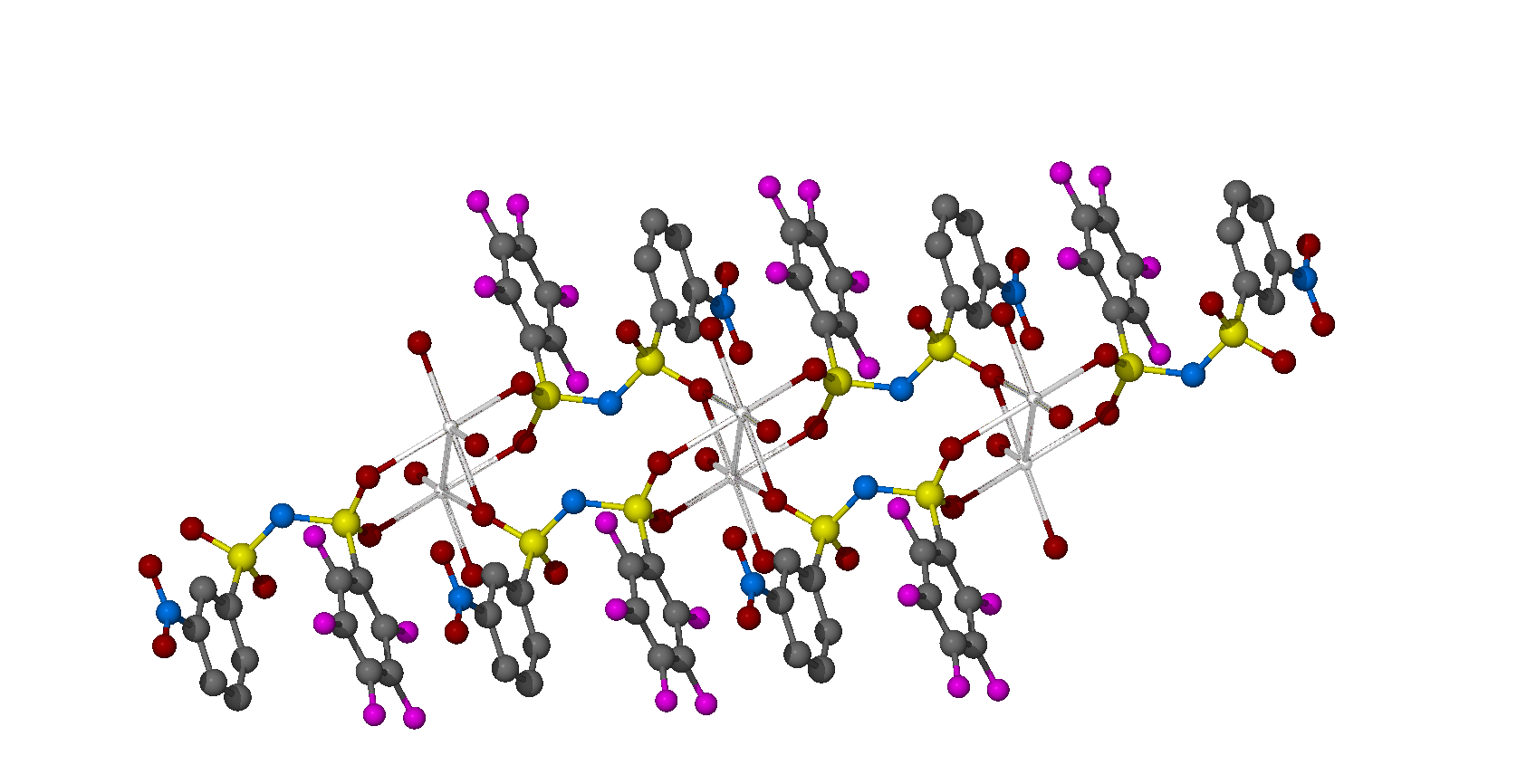


Figure S6: Ball and stick representation of the X-ray crystallographic structure of **3** that showcases the coordination to sodium ions by ligand molecules in lattice. Atom labelling: carbon (black), oxygen (red), nitrogen (blue), fluorine (pink), sulphur (yellow) and sodium (silver).

**4** crystallised in the P-1 space group and was isolated as the sodium salt. In the crystal structure, two different types of sodium ion are present. All together three sodium ions are present in the asymmetric unit with a total occupancy of 2.5. The unit cell is shown in Figure S7; five sodium ions are present. Two sodium ions both have fully occupancy in the asymmetric unit with each ion being coordinated to four different ligands through an oxygen atom of each sulphonate ligand. Each sodium ion is in a distorted octahedral environment (*trans* O-Na-O bond angles are 174.06°, 160.78° and 171.10°). The remaining two coordination sites are occupied by two water molecules. Each water molecule bridges two sodium ions (bond angles for Na-O-Na are 116.20° and 116.63°). The hydrogen atoms for the bridging water molecules could not be found in the difference map.

The second type of sodium ion only has half occupancy. Again, it is coordinated to four different ligands through the oxygen atoms from a sulphonate group and to two water molecules. Unlike compound **3**, the water molecules are disordered over two positions with a 52%:48% probability of being in either position. Again, the H-atoms could not be located for these oxygen atoms from the difference map. One of the nitrogen atoms that is adjacent to two sulphonate groups is also disordered over two positions with exactly the same probability as the water molecule oxygen atoms Furthermore, the oxygen atoms of one of the sulphonate groups is also disordered.


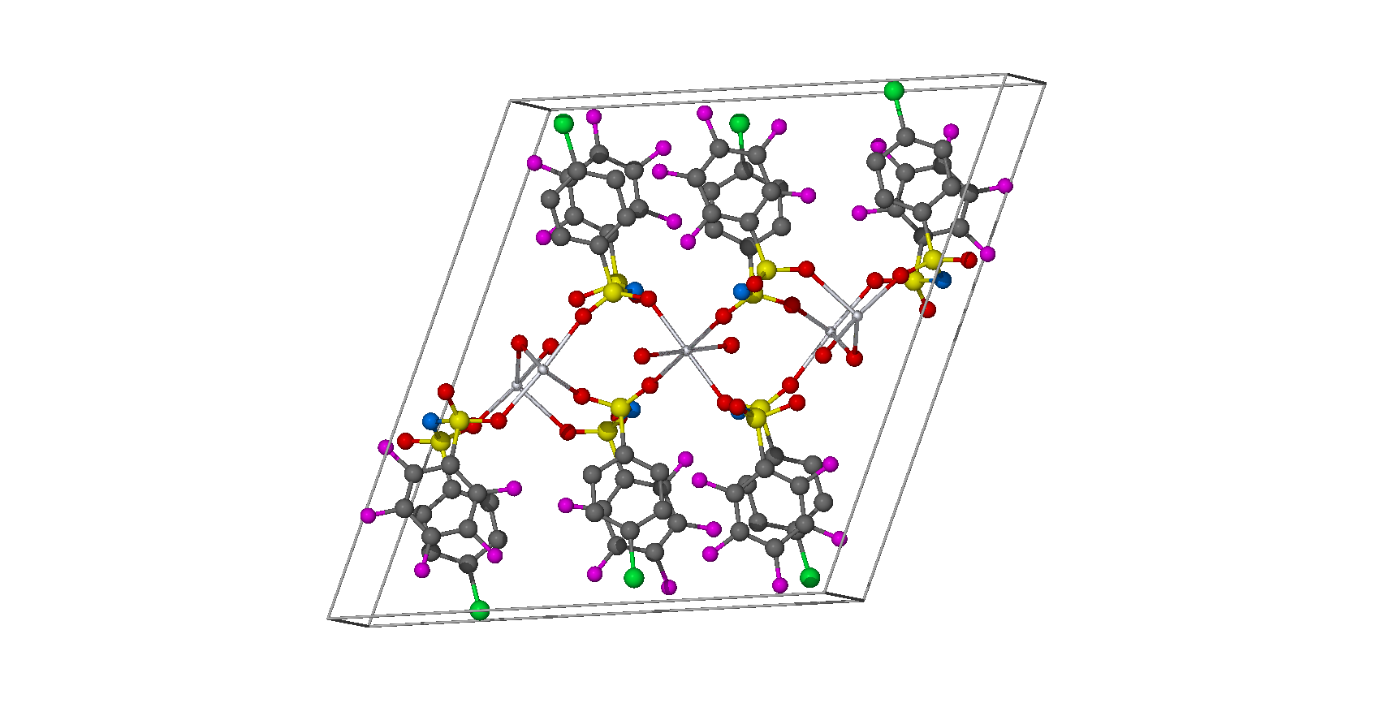


Figure S7: Ball and stick representation of the X-ray crystallographic unit cell contents of **4** with H atoms omitted for clarity. For atoms that are disordered over two positions, only one position is shown. The sodium ion in the centre of the unit cell has half occupancy compared to the other four sodium ions shown. Atom labelling: carbon (black), oxygen (red), nitrogen (blue), fluorine (pink), sulphur (yellow) and sodium (silver).

The ligands possess π-π stacking which is evident along the b-c plane (Figure S8). The centroid-to-centroid distance between the two phenyl rings of the ligands reveal that the closest contact is 3.539 Å (symmetry code = x, y, z). The same contact distance links centroids of adjacent rings of symmetry-linked elements. This π-π distance is very similar to that observed for **3**.


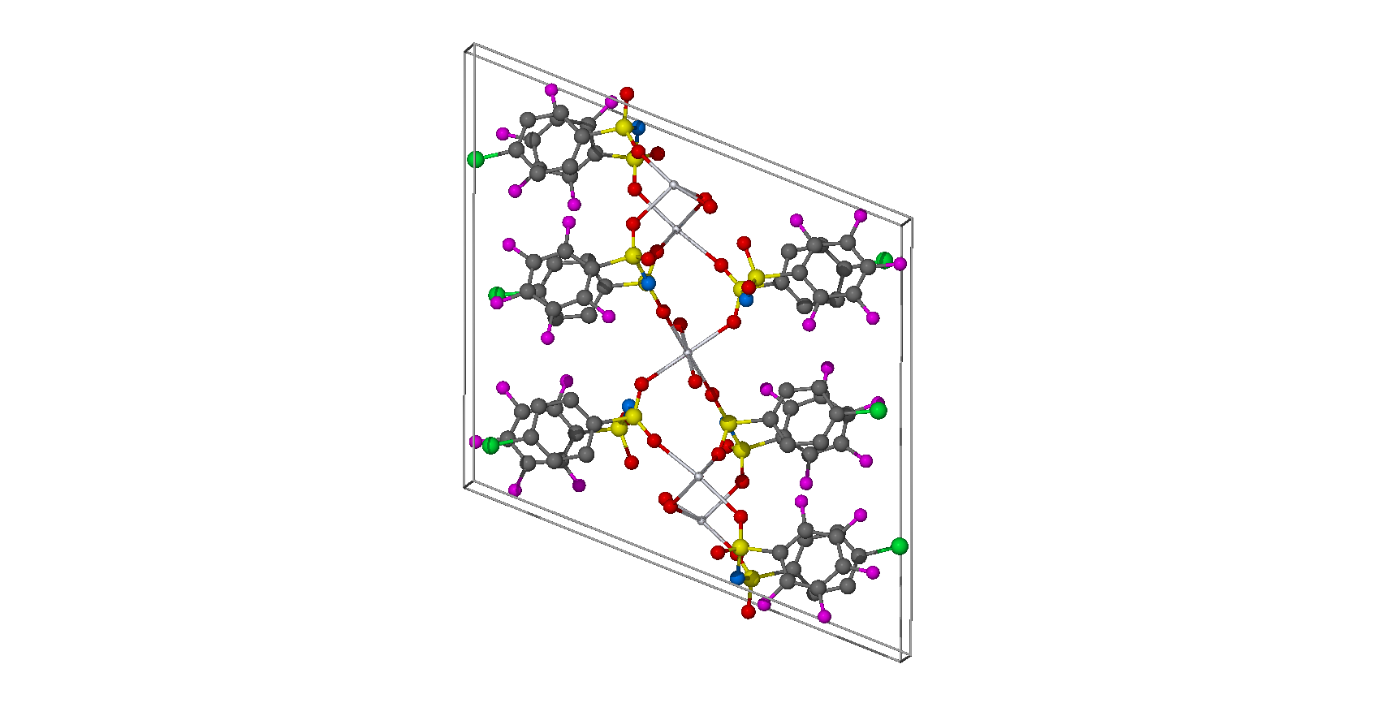


Figure S8: Ball and stick representation of the X-ray crystallographic unit cell contents of **4** viewed along the b-c plane. H atoms have been omitted for clarity. For atoms that are disordered over two positions, only one position is shown. Atom labelling: carbon (black), oxygen (red), nitrogen (blue), fluorine (pink), sulphur (yellow) and sodium (silver).

Table S3**:** Crystallographic data for compounds *rac*-**1**, **3**-**5** and **7**.

| Compound number | **rac-1** | **3** | **4** | **5** | *7* |
| --- | --- | --- | --- | --- | --- |
| Formula | C_22_H_13_F_4_O_2_P | C_24_H_18_N_4_O_16_F_10_Na_2_S_4_ | C_72_H_24_Cl_6_F_30_N_6_Na_5_O_30_S_12_ | C_22_H_18_ | C_24_H_17_F_4_O_2_P |
| M_,_ g mol^-1^ | 416.29 | 982.64 | 2735.34 | 282.39 | 444.34 |
| Crystal system | Monoclinic | Triclinic | Triclinic | Monoclinic | Monoclinic |
| Space group | *P_21_/_c_* | *P-1* | *P-1* | *C_2_/c* | *P_21_/_c_* |
| a/Å | 10.999(2) | 7.2301(2) | 7.4286(2) | 17.0224(3) | 15.186(3) |
| b/Å | 12.705(2) | 8.0035(2) | 17.9262(7) | 7.4345(10) | 18.106(3) |
| c/Å | 25.642(5) | 33.1658(8) | 19.3116(8) | 24.9292(4) | 14.814(2) |
| α/deg | 90 | 86.251(2) | 111.638(4) | 90 | 90 |
| β/deg | 95.21(3) | 86.228(2) | 92.039(3) | 101.364(2) | 94.653(3) |
| γ/deg | 90 | 63.409(2) | 92.300(3) | 90 | 90 |
| V/Å^3^ | 3568.6(12) | 1711.07(8) | 2385.00(16) | 3093.02(9) | 4060.0(11) |
| T/K | 173(2) | 293(2) | 293(2) | 293(2) | 173(2) |
| Z | 8 | 2 | 1 | 8 | 8 |
| ρ_, calc_ [g cm^-3^] | 1.550 | 1.9070 | 1.904 | 1.2127 | 1.454 |
| λ^c^/ Ǻ | 0.71073 | 1.54184 | 1.54184 | 1.541840 | 0.71073 |
| Data Measured | 24019 | 27410 | 39761 | 26663 | 8228 |
| Ind. Reflns | 8117 | 6761 | 9274 | 3106 | 8228 |
| R_int_ | 0.0329 | 0.0734 | 0.0667 | 0.0291 | 0.0355 |
| Reflns with I  I > 2σ(I) | 6300 | 5830 | 7384 | 2825 | 6430 |
| Parameters | 529 | 565 | 774 | 89 | 561 |
| Restraints | 0 | 8 | 31 | 0 | 0 |
| R_1_^d^ (obs), wR_2_^d^ (all) | 0.0451, 0.0605 | 0.0686, 0.0774 | 0.1042, 0.1262 | 0.0709, 0.0753 | 0.0579, 0.0731 |
| Goodness of fit | 1.053 | 1.129 | 1.064 | 1.058 | 1.048 |
| Largest residuals/ e Ǻ ^-3^ | 0.880, −0.387 | 1.344, −0.718 | 2.571, −2.041 | 0.607, −0.415 | 0.868, −0.776 |

^c^MoK utilised for **1** and **7**, Cu/K for **3**-**5**.

^d^*R*1 =Σ||*F*_o_| - |*F*_c_||/Σ|*F*_o_|, *wR*2 = *{*Σ[*w*(*F*_o_^2^-*F*_c_^2^)^2^]/Σ[*w*(*F*_o_^2^)^2^]*}*^1/2^.

# References

1. Oxford Diffraction, *CrysAlisPro*, 2006, Oxford Diffraction Ltd, Abingdon, Oxfordshire, England.

2. G. M. Sheldrick, *SHELXS97 and SHELXL97. Program for Crystal Structure Solution and Refinement*, 1997, University of Göttingen, Göttingen.

3. K. Fujii, H. Todani, S. Ito and K. Mikami, *Org. Lett.*, 2019, **21**, 3387-3391.

4. I. Leito, E. Raamat, A. Kütt, J. Saame, K. Kipper, I. A. Koppel, I. Koppel, M. Zhang, M. Mishima, L. M. Yagupolskii, R. Y. Garlyauskayte and A. A. Filatov, *J. Phys. Chem. A*, 2009, **113**, 8421-8424.

5. N. Pala, L. Micheletto, M. Sechi, M. Aggarwal, F. Carta, R. McKenna and C. T. Supuran, *ACS Med. Chem. Lett.*, 2014, **5**, 927-930.

6. A. Kütt, S. Tshepelevitsh, J. Saame, M. Lõkov, I. Kaljurand, S. Selberg and I. Leito, *Eur. J. Org. Chem.*, 2021, **2021**, 1407-1419.

7. R. Shivapurkar and D. Jeannerat, *Anal. Methods*, 2011, **3**, 1316-1322.

8. I. Fujii and N. Hirayama, *Helv. Chim. Acta*, 2002, **85**, 2946-2960.

9. R. A. Aitken, R. A. Inwood and A. M. Z. Slawin, *J. Chem. Crystallogr.*, 2021, **51**, 497-504.
